# MCAM is a novel metastasis marker and regulates spreading, apoptosis and invasion of ovarian cancer cells

**DOI:** 10.1007/s13277-012-0417-0

**Published:** 2012-05-19

**Authors:** Zheng Wu, Zhiyong Wu, Jun Li, Xiaomei Yang, Yahui Wang, Yi Yu, Jun Ye, Congjian Xu, Wenxin Qin, Zhigang Zhang

**Affiliations:** 1 Shanghai Medical College of Fudan University, No.138 Yi-Xueyuan Road, Shanghai, 200032 China; 2 State Key Laboratory of Oncogenes and Related Genes, Shanghai Cancer Institute, Renji Hospital, Shanghai Jiao Tong University School of Medicine, No. 25 Xie-tu Road, Shanghai, 200032 China; 3 Department of Gynecology, Obstetrics and Gynecology Hospital, Fudan University, No. 419 Fang-Xie Road, Shanghai, 200011 China; 4 Shanghai 5th People’s Hospital Medical Center of Fudan University, No. 128 Rui-Li Road, Shanghai, 200240 China

**Keywords:** MCAM, Ovarian cancer, Spreading, Invasion, Apoptosis

## Abstract

Melanoma cell adhesion molecule (MCAM) is a cell adhesion molecule that is abnormally expressed in a variety of tumours and is closely associated with tumour metastasis. The role of MCAM in ovarian cancer development has not been fully studied. In this study, through immunohistochemical staining of ovarian cancer tissue samples and RNA interference to silence MCAM in ovarian cancer cells, we examined the impact of MCAM on the biological functions of ovarian cancer cells and attempted to reveal the role of MCAM in ovarian cancer development. Our results showed that MCAM expression was particularly high in metastatic ovarian cancers compared with other pathological types of ovarian epithelial tissues. After MCAM silencing in the MCAM high-expression ovarian cancer cell line SKOV-3, the cell apoptosis was increased, whereas the cell spreading and invasion were significantly reduced, which may be related with dysregulation of small RhoGTPase (RhoA and Cdc42).These results suggest that MCAM expression in ovarian cancer is highly correlated with the metastatic potential of the cancer. MCAM is likely to participate in the regulation of the Rho signalling pathway to protect ovarian cancer cells from apoptosis and promote their malignant invasion and metastasis. Therefore, MCAM can be used not only as a molecular marker to determine the prognosis of ovarian cancer but also as a therapeutic target in metastatic ovarian cancer.

## Introduction

Ovarian cancer has become one of the primary tumours that pose a serious threat to women's lives and health globally. Although the incidence rate is not high, the death rate is the highest among all gynaecological tumours [1]. The major cause of death in advanced ovarian cancer is metastasis, which is a complex process that involves changes in many molecules, including adhesion molecules, proteolytic enzymes, chemokines and so on. Among them, the adhesion molecules between cell–cell and cell matrix have drawn much attention in cancer metastasis research [2].

It is generally believed that the lack of function of cell adhesion molecules will facilitate tumour cell dissemination. For example, in epithelial ovarian cancer, opioid-binding cell adhesion molecule is often inactivated by allelic deletion or by methylation [3]. In addition, the down-regulation of CD9 indicates a poor prognosis because this change can cause a reduction in the expression of certain integrins, thus leading to the metastasis of ovarian cancer [4]. Interestingly, the elevated expression of certain other adhesion molecules, such as *p*-cadherin, can promote ovarian cancer metastasis [5]. Therefore, the role of adhesion molecules in the metastasis of ovarian cancer is complex and requires further study.

A member of the immunoglobulin superfamily, melanoma cell adhesion molecule (MCAM; also known as CD146 or MUC18) was first identified in melanoma [6]. MCAM is a membrane calcium-independent glycoprotein adhesion molecule, the extracellular domain of which contains the typical V-V-C2-C2-C2 Ig-like domain and the intracellular structure of which contains several protein kinase recognition motifs, suggesting that MCAM may participate in cell signalling pathways inside and outside the cell [7]. MCAM was initially considered to be the characteristic antigen that distinguishes malignant melanoma from benign or borderline melanoma. Follow-up studies found that MCAM is abnormally expressed in a variety of tumour tissues, including melanoma [8], prostate cancer [9], breast cancer [10] and non-small cell lung cancer [11] and that this abnormal expression is closely associated with tumour progression and metastasis. In 2006, Aldovini et al. reported that epithelial ovarian cancer patients with high expression of MCAM in tumour tissues had a significantly higher relapse rate than MCAM expression-negative patients and that the survival period of the former group was significantly shorter [12].

In this study, we have found that borderline ovarian tumours and malignant epithelial ovarian cancer have higher MCAM-positive rates compared with normal ovarian epithelium and benign ovarian tumours. The MCAM expression rate is particularly high in metastatic ovarian cancer lesions. We further used RNA interference to silence MCAM gene expression in the ovarian cancer cell line SKOV-3, and our results showed that, after MCAM knockdown, the cancer cell apoptosis was increased, and the capacities of cell spreading on the extracellular matrix and invasion through matrigel were significantly reduced. The down-regulation of MCAM expression was also correlated with decreased Rho GTPases (Cdc42 and RhoA) activation. Our study has demonstrated that MCAM affects ovarian cancer cell apoptosis and invasion, indicating that, in addition to being used as a molecular marker to determine the prognosis of ovarian cancer, MCAM may also be used as a new target for clinical treatment.

## Materials and methods

### Cell culture and chemical reagents

Ovarian cancer cell lines SKOV-3 (purchased from the Cell Bank of the Chinese Academy of Science, Shanghai, China.), OVCA429 and RMUG-S (gifts from prof. Bin Ye, Harvard Medical School, Boston, MA ) were cultured at 37 °C in a humidified 5 % CO_2_ atmosphere in RPMI-1640 medium with 10 % fetal calf serum (Gibco, Invitrogen, Carlsbad, CA), 100 IU/ml penicillin G, and 100 mg/ml streptomycin sulfate (Sigma-Aldrich, St. Louis, MO). X-tremeGENE siRNA Transfection Reagent (Roche Diagnostics GmbH, Roche Applied Science, Mannheim, Germany) and Opti-MEM–1 Medium (Gibco, Invitrogen, Carlsbad, CA) were used for siRNA transfection. The siRNAs were synthesised by Shanghai GenePharma Co. Rabbit polyclonal antibodies used in this study were directed against MCAM (ProteinTech Group, Inc (Chicago, IL). Rabbit monoclonal antibodies used in this study were directed against RhoA and Cdc42 (Cell Signalling Technology, Inc., Danvers, MA). Mouse monoclonal antibodies used in this study were directed against Rac1 (Merck Millipore, Danvers, MA), tubulin (Sigma, St. Louis, MO) and Ki67 (Abcam, Hong Kong). IRDye 680/800 conjugated second antibodies were from LI-COR, Inc. (Lincoln, NE). Collagen I was purified in our laboratory [13], Collagen IV, fibronectin and laminin 1 were purchased from Merck Millipore (Danvers, MA). Cell Counting Kit8 (CCK8) was a product of Dojindo Molecular Technologies, Inc. (Kumamoto, Japan). In Situ Cell Death Detection Kit was from Roche Applied Science (Mannheim, Germany)and FITC Annexin V Apoptosis Detection Kit was from BD Biosciences (San Jose, CA).

### Clinical samples and immunohistochemical staining

Human ovary tissue microarrays (OV1005a and OV808) contained 45 cases of primary malignant epithelial ovarian cancer, 40 metastatic ovarian cancer, 7 borderline cystadenoma, 16 benign cystadenoma, 17 cancer adjacent normal ovary tissues and 3 normal ovary tissues were purchased from US Biomax Inc (Alenabio, Xi’an, China). Immunohistochemistry was performed for MCAM according to standard procedures as described [14]. All of the sections were observed and photographed with a microscope (Axio Imager.A1, Carl Zeiss MicroImaging GmbH, Germany). After nuclear counterstaining with hematoxylin, the cytoplasmic and cytomembrane of epithelial cells immunostaining intensity was categorised semiquantitatively into four groups: negative (score 0): no staining at all, weakly positive (score 1): faint/barely perceptible staining in the majority of the epithelial cells, moderately positive (score 2): a moderate staining in the majority of the tumour cells, and strongly positive (score 3): a strong staining of the majority of the tumour cells. The final score was designated as negative or positive as follows: score of 0–1, negative, score of 2–3, positive. These scores were determined independently by two senior pathologists.

### Quantitative real-time PCR

Total RNA extracted using Trizol reagent (TaKaRa, Japan), and reversely transcribed through PrimeScript RT-PCR kit (TaKaRa, Japan) according to the protocol. Real-time PCR analyses were performed with SYBR *Premix Ex Taq* (TaKaRa, Japan) on a 7300 Real-time PCR system (Applied Biosystems, Inc. USA) at the recommended thermal cycling settings: one initial cycle at 95 °C for 10 s followed by 40 cycles of 5 s at 95 °C and 31 s at 60 °C. Primer sequences used for MCAM detection were as follows, sense: 5′-GGGTACCCCATTCCTCAAGT-3′ and antisense: 5′-CCTGGACTCCTTCATGTGGT-3′ [15]. The expression level were normalised to the internal reference gene 18s rRNA (sense, 5′-GTAACCCGTTGAACCCCATT-3′; antisense, 5′-CCATCCAATCGGTAGTAGCG-3′) [16].

### Western blotting and GTPase pull-down assays

Cells were lysed in lysis buffer(50 mM Tris–HCl, 150 mM NaCl, 1 % Triton-X 100, 1 Mm each MgCl_2_, MnCl_2_ and CaCl_2_, 1 mM PMSF and 10 mM sodium fluoride), then mixed with Laemmli buffer. Proteins were separated by SDS-PAGE under reducing condition, followed by immunoblotting with specific primary antibodies (anti-MCAM and anti-tubulin) and species-specific secondary antibodies. Bound secondary antibodies were revealed by Odyssey imaging system (LI-COR Biosciences, Lincoln, NE). GTPase pull-down assays were performed according to standard procedures as described [17].

### siRNA transfection

Small interfering RNAs duplexes for MCAM were as follows: MCAM-si1 sense, 5′-GACUUGGACACCAUGAUAUTT-3′, anti-sense, 5′-AUAUCAUGGUGUCCAAGUCTT-3′; MCAM-si2 sense, 5′-GGUGUUGAAUCUGUCUUGUTT-3′, anti-sense, 5′-ACAAGACAGAUUCAACACCTT-3′. Transfection steps were following the manufacture’s protocols.

### Cell proliferation assay and apoptosis assay

Cell proliferation Assay was tested with the CCK8 Assay. And cell death was detected by Direct TUNEL labeling assay or flow cytometric analysis of FITC Annexin V staining. All processes were according to the manufacture’s protocols.

### Cell invasion assay

Seventy microlitres of 1:6 diluted Matrigel (2–3 mg/ml protein) was added into the centre of each chamber (Merck Millipore, Danvers, MA) laid in the 24 wells plate (Corning, NY). After coating in incubator for 20–30 min, 1 × 10^5^ cells in 150 μl of defined medium were plated into upper chamber, with 600 μl of medium to the lower chamber. After culturing for approximately 48 h, the cells were fixed with 0.5 ml of 1 % glutaraldehyde in 1× PBS. Then washed each well three times with 1× PBS, and stained with 0.6 ml of 0.5 % crystal violet solution. After removing cells on the upper chamber using a cotton swab, counted the number of cells at five fields per membrane with the microscope (Axio Imager.A1).

### Cell adhesion and spreading assay

Assays were performed as described previously by Zhang et al. [18]. The area of spreading cells’ surface was measured by an image software, Image-Pro Plus 6.0 (Media Cybernetics, Inc., Bethesda, MD). And in each group, at least 50 adherent cells were calculated.

### Statistical analysis

The results were presented as the means and SDs. The data was subjected to Student’s *t*-test (two tailed; *p* < 0.05 was considered significant) and *χ* ^2^ test was used to analyse the distribution of MCAM-positive cases in relation to clinical and pathology category variables.

## Results

### MCAM expression varies among different pathological types of ovarian epithelial tissues

**Fig. 1:**
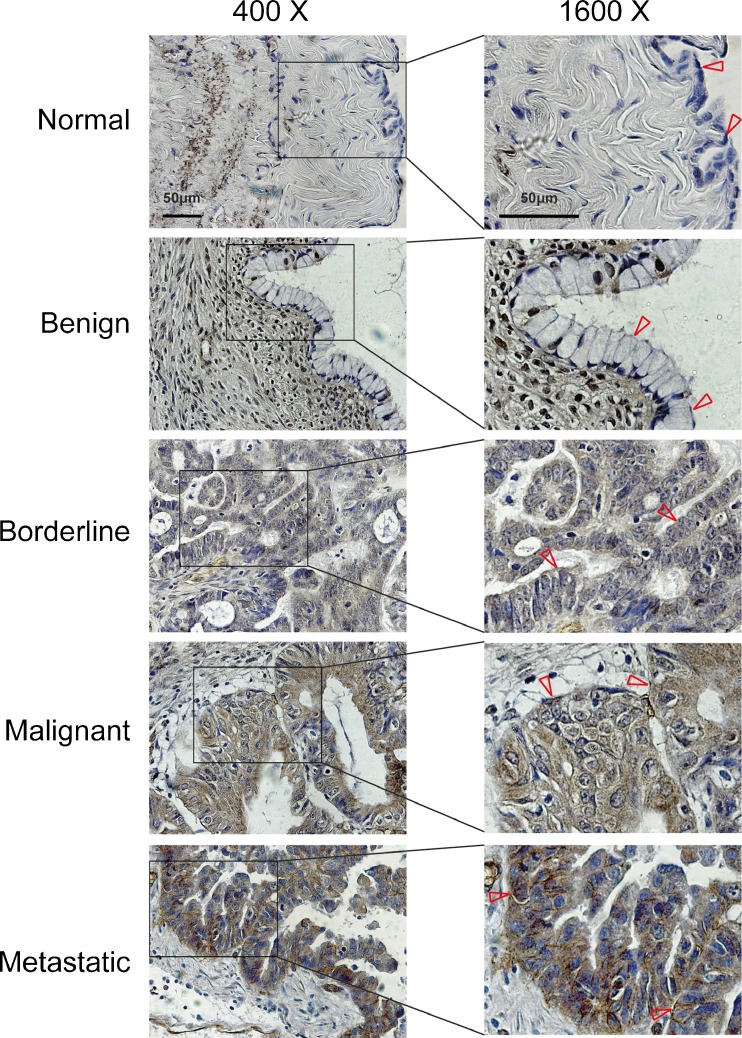
Immunohistochemical analysis of MCAM expression in normal ovarian epithelial tissue, benign, borderline, malignant and metastatic ovarian tumour tissues. *Left panels*, original magnification, ×400. *Right panels*, ×4 magnification of area indicated on the *left*. The epithelium is denoted by *arrows*

The MCAM expression levels of different pathological types of ovarian epithelial tissues were examined by immunohistochemical methods (Fig. 1). Further statistical analysis showed that MCAM expression was positive in three cases (15.79 %) among 19 cases of normal and benign tumour tissue, 21 cases (46.67 %) of malignant epithelial ovarian cancer showed positive expression among 45 cases examined, 6 cases (85.71 %) of borderline ovarian tumours showed positive expression among 7 cases examined, and 32 cases (80.00 %) of metastatic disease tissues showed positive expression among 40 cases examined. The MCAM-positive rate increased in malignant epithelial ovarian cancers compared with normal and benign tissues significantly (*p* = 0.020). The MCAM-positive rate in the metastatic tumour tissue was extremely higher than in the normal ovarian epithelial tissue and benign tumours (*p* < 0.001) and was observably different from that in the malignant ovarian tumour tissues (*p* = 0.002). No significant correlation was detected between MCAM expression and ovarian cancer grading, stage, or patient age (Table 1).

**Table 1:** Patient’s clinical and pathological characteristics and their association with MCAM expression

MCAM expression (*n* = 111)				
	Cases	Positive^b^	Negative^b^	*p* ^c^
Normal and benign	19	3 (15.79)	16 (84.21)	
Borderline	7	6 (85.71)	1 (14.29)	0.001 (versus normal)
Carcinoma	45	21 (46.67)	24 (53.33)	0.020 (versus normal)
Metastasis	40	32 (80.00)	8 (20.00)	<0.001 (versus normal)
				0.002 (versus carcinoma)
Stage				
I–II	34	16 (47.06)	18 (52.94)	
III–IV	11	6 **(**54.55)	5 (45.45)	0.062
Grading				
1	21	14 (66.67)	7 (33.33)	
2	41	28 (68.29)	13 (31.71)	0.548 (versus grading 1)
3	23	15 (65.22)	8 (34.78)	0.514 (versus grading 1)
Age^a^				
<Mean	42	29 (69.05)	13 (30.95)	
≥Mean	43	26 (60.47)	17 (39.53)	0.408

^a^Average age of all cases was 50 years old (range from 22 to 75)

^b^Values in parentheses indicate percentage values

^c^Evaluated by *χ* ^2^ test

### Knock-down MCAM in ovarian cancer cell lines

**Fig. 2:**
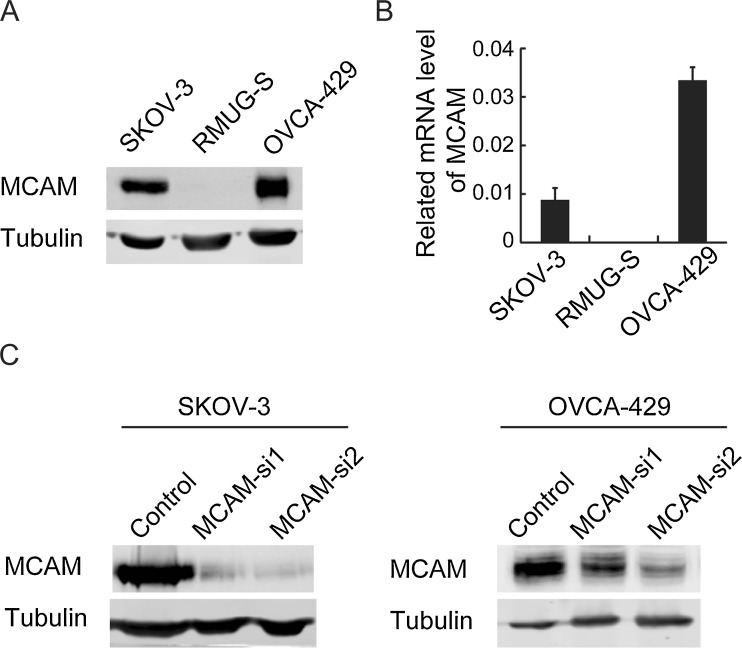
Expression of MCAM in ovarian cancer cell lines and knock-down of MCAM in ovarian cancer cells. **a** Lysates from SKOV-3, RMUG-S and OVCA-429 cells were subjected to SDS-PAGE, followed by immunoblotting for MCAM. Tubulin was used as loading control. **b** The relevant mRNA levels of MCAM in these three cell lines were tested by quantitative real-time PCR, which were normalised to 18s rRNA. **c** Western blot analysis of lysates from SKOV-3 and OVCA-429 cells at 48-h after transfection of one of the two different MCAM siRNAs or of negative control siRNA. The cells were analysed by immunoblotting with specific antibodies to MCAM

Reverse-transcription polymerase chain reaction and Western blotting analyses were used to examine three human ovarian cancer cell lines. The results showed that MCAM expression was higher in the SKOV-3 and OVCA-429 cell lines, whereas MCAM expression was almost absent in the RMUG-S cells (Fig. 2a, b). We therefore chose SKOV-3 and OVCA-429 cells for further experiments. Two siRNAs specific for MCAM and a negative control siRNA were transfected into SKOV-3 and OVCA-429 cells, and the change in the MCAM expression level was analysed 48 hours later. As shown in Fig. 2c, the MCAM protein levels decreased significantly after transfection with siRNA1 in SKOV-3 cell and siRNA2 in both cells.

### Silencing of MCAM induced apoptosis of ovarian cancer cell

**Fig. 3:**
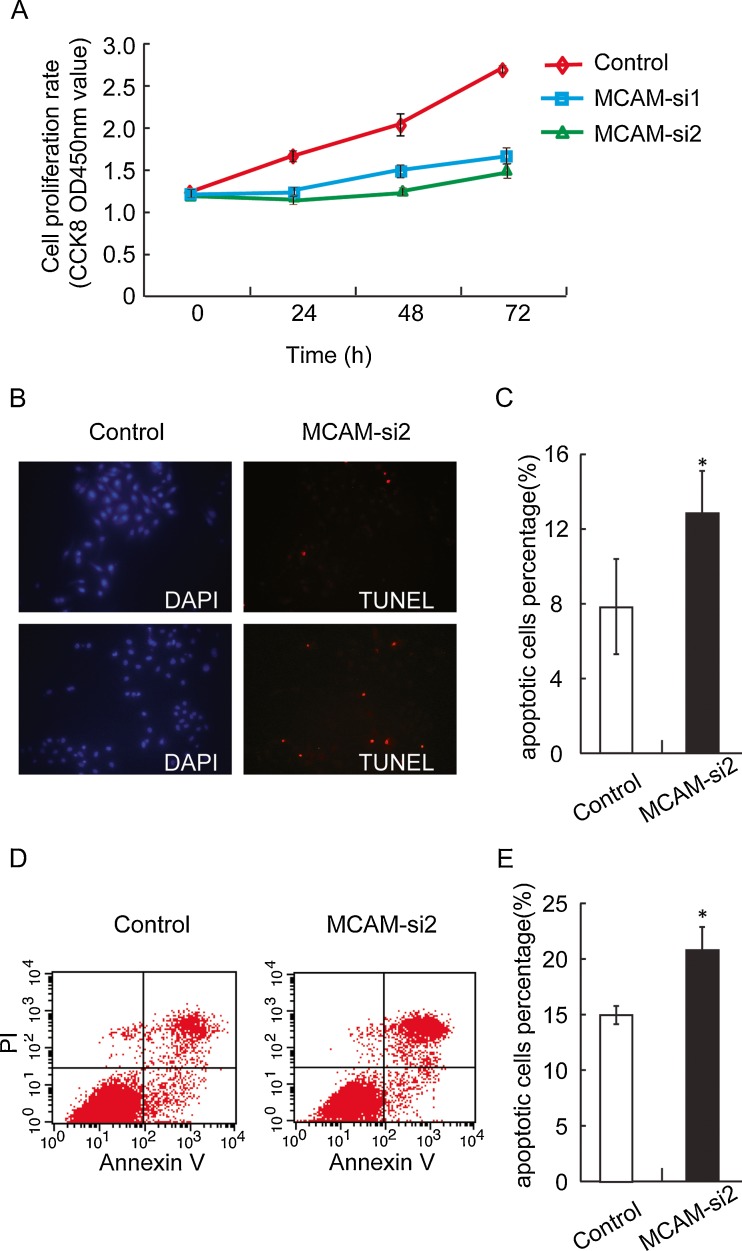
Effects of MCAM knock-down on ovarian cancer cells growth and apoptosis. **a** Equal amount of cells were seeded initially and the number of viable SKOV-3 cells at different time points were determined by CCK8 assay for up to 72 h (mean±SD, *n* = 3). **b** TUNEL staining to detect DNA fragmentation in SKOV-3 cells. *Left panel*, cells were transfected with negative control siRNA; *right panels*, transfected with MCAM siRNA-2. **c** Quantification of TUNEL-positive SKOV-3 cells. **d**, **e** Flow cytometric analysis using Annexin V to detect apoptotic OVCA-429 cells. Independent *t* tests were used for all statistical comparisons. After TUNEL and DAPI staining, the cells were counted in ten fields. The percentage of TUNEL-positive cells divided by the number of blue cells in a given field was determined and average of different fields is presented (*p < 0.05)

Previous studies have found that MCAM over-expression can promote tumourgenesis and growth of melanoma cells in nude mice [8]. Our results showed that, 24 h after transfection, the growth rate of SKOV-3 cells transfected with MCAM siRNA was significantly lower than that of the negative control group (Fig. 3a). To find out the reason of changes in growth rate after silencing of MCAM, we examined cell apoptosis with TUNEL assay and flow cytometric analysis, The apoptotic rate of MCAM-si2-SKOV-3 group was 12.64 % ± 2.40 %, significantly higher than that of control group (7.97 % ± 2.51 %) (Fig. 3b, c). And the apoptotic rate of MCAM-si2-OVCA-429 group was 20.68 % ± 1.85 %, also higher than that of control group (14.97 % ± 0.75 %) (Fig. 3d, e). In contrast, the proliferation indices had no difference between the two groups assessed by nuclear localisation of Ki67 (data not shown).

### MCAM silencing inhibited in vitro invasion of ovarian cancer cells

**Fig. 4:**
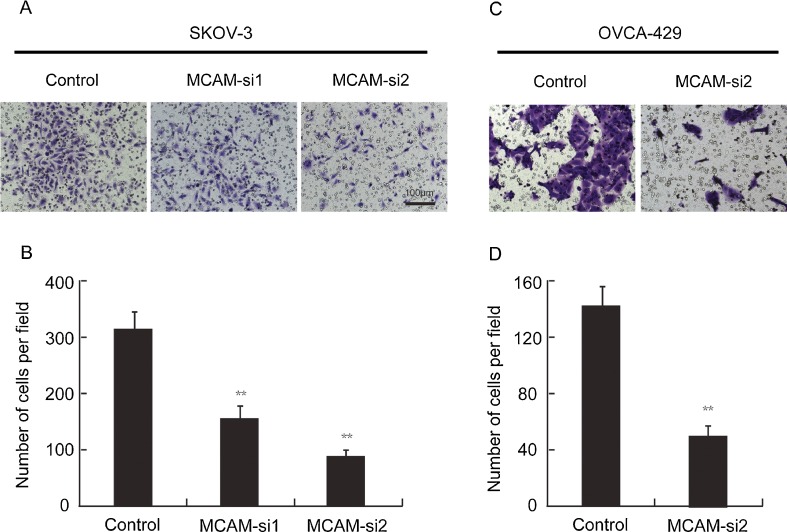
Silencing of MCAM inhibits ovarian cancer cells invasion in vitro. **a**, **c** Cells that have invaded through the Matrigel and onto the lower surface of the porous membrane are stained *purple*. **b**, **d** Quantification of cells on the bottom surface of the membrane from independent assays performed in triplicate (means indicated). There were fewer invaded cells in the MCAM siRNA groups than in the negative control siRNA group (**p < 0.01 when compared with the control group)

To investigate the impact of MCAM on invasion of SKOV-3 and OVCA-429 cells, we performed an in vitro invasion assay. As shown in Fig. 4a, the number of SKOV-3 cells passing through the Matrigel in the negative control group (318 ± 31) was significantly higher than that in the MCAM-si1 (153 ± 23) and MCAM-si2 group (88 ± 8). And the number of OVCA-429 cells passing through the Matrigel per field in the negative control group (142 ± 14) was significantly higher than that in the MCAM-si2 (50 ± 6). These results suggest that MCAM knockdown can significantly reduce the in vitro invasion ability of ovarian cancer cells.

### Silencing of MCAM decreased the ability of ovarian cancer cells to spread on extracellular matrix proteins

**Fig. 5:**
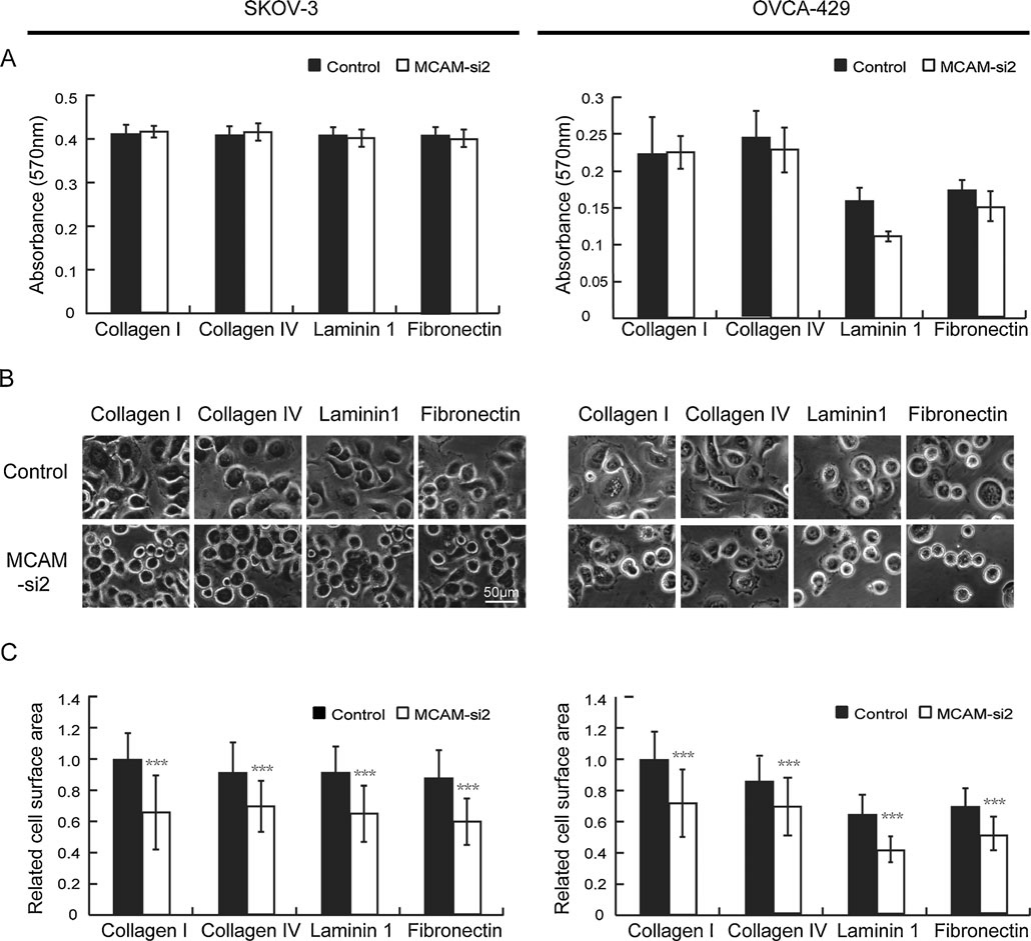
Adhesion and spreading of MCAM-silenced and control ovarian cancer cells on extracellular matrix proteins. **a** Adhesion of MCAM-silenced and control SKOV-3 and OVCA-429 cells to extracellular matrix proteins. Equal numbers of control (*black columns*) and MCAM-si2 (*gray columns*) cells were seeded in triplicate wells coated with optimal concentrations of collagen I (10 mg/ml), collagen IV (10 mg/ml), laminin 1 (10 mg/ml) and fibronectin (10 mg/ml). The extent of cell adhesion to the different substrates was measured after 30 min by crystal violet staining and color readings. The mean absorbance of triplicate wells and SDs were shown. **b** Spreading of MCAM silencing and control cells on extracellular matrix proteins. After 30 min of adhesion to ECM protein, MCAM silencing and control cells were stained with crystal violet and photographed under phase contrast microscopy. Bar, 50 μm. **c** Area of spread cells’ surface was quantified by Image-Pro Plus 6.0 (Media Cybernetics, Inc., MD) in a population of at least 50 adherent cells on each group. Bars indicate SD. Statistically significant differences are indicated: ***p < 0.0001

The level of cell spreading on extracellular matrix proteins reflects the ability of interaction between the cells and matrix. It has been reported that, by regulating integrin, MCAM can indirectly affect the cells’ adhesion ability to the extracellular matrix protein laminin 1 [19]. We examined the adhesion and spreading of the SKOV-3 and OVCA-429 cells to four matrix proteins (collagen I, collagen IV, laminin 1 and fibronectin) after silencing of MCAM. The adhesion of MCAM-silenced cells to all four tested substrates is similar to that of control cells (Fig. 5a) while the cell spreading on the four tested substrates was significantly different between the two groups. After 30-min adhesion, the control group cells showed a well spread and flat morphology, whereas the MCAM konckdown cells were mostly rounded (Fig. 5b). The relative spreading areas of SKOV-3 cells in the negative control group on the four matrix proteins were 1 ± 0.17 (collagen I), 0.9 ± 0.2 (collagen IV), 0.91 ± 0.18 (laminin 1), and 0.88 ± 0.19 (fibronectin), and the relative spreading areas of the MCAM knockdown group were 0.66 ± 0.24 (collagen I), 0.69 ± 0.16 (collagen IV), 0.64 ± 0.18 (laminin 1) and 0.60 ± 0.15 (fibronectin). While the relative spreading areas of OVCA-429 cells in the negative control group on the four matrix proteins were 1 ± 0.18 (collagen I), 0.84 ± 0.15 (collagen IV), 0.62 ± 0.12 (laminin 1) and 0.67 ± 0.11 (fibronectin), and the relative spreading areas of the MCAM knockdown group were 0.70 ± 0.21 (collagen I), 0.67 ± 0.18 (collagen IV), 0.40 ± 0.08 (laminin 1) and 0.49 ± 0.11 (fibronectin) (Fig. 5c). Therefore, MCAM silencing mainly affected the ability of ovarian cancer cells to spread in the matrix protein.

### MCAM participates in the regulation of the Rho GTPase signalling pathway

**Fig. 6:**
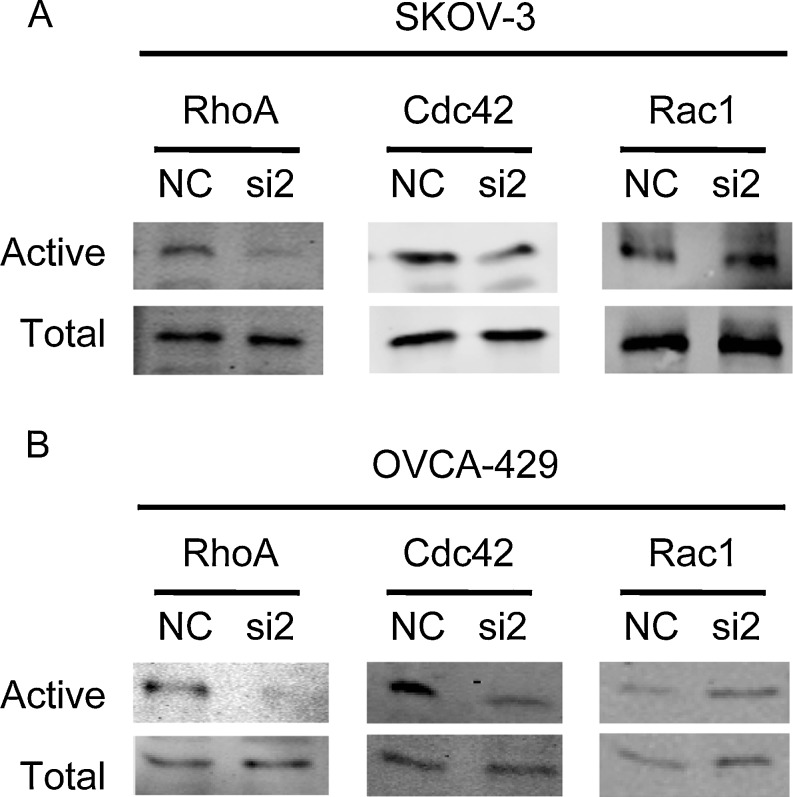
Dysregulation of Rho GTPases activities after silencing of MCAM. SKOV-3 (**a**) and OVCA-429 (**b**) cells were serum starved for 24 h and the activity of RhoA, Cdc42 and Rac1 was measured by pull-down assays in MCAM siRNA2-transfected cells and negative control cells. Signals obtained for the pull-down samples correspond to 800 ml of lysate for the blots with anti-RhoA and anti-Cdc42 and 500 ml of lysate for the blot with anti-Rac1. Aliquots of the respective lysates (40 ml) were used to analyse the total amount of each GTPase (NC denoted negative control; si2 denoted MCAM-si2)

Rho GTPase plays an important role in regulating actin polymerisation, myosin contraction, cell adhesion, and microtubule dynamics, thereby regulating cell shape, polarity and movement. These traits are necessary for tumour cell invasion and metastasis. A large number of studies have shown that the Rho signalling pathway is involved in the occurrence and development of malignant tumours and that its over-expression and increase in activity are closely associated with the invasion and metastasis of malignant tumours. We examined Rho GTPase activity after MCAM knockdown in SKOV-3 and OVCA-429 cells. Pull-down assay results showed that the down-regulation of MCAM expression decreased the amount of GTP-bound RhoA and Cdc42, and the activity of Rac1 was not altered (Fig. 6).

## Discussion

Invasion and metastasis are complex pathological processes that involve not only interactions among tumour cells and interactions between tumour cells and host cells but also the complex regulation of many molecules. Changes in the expression of cell adhesion molecule (CAMs) have been confirmed in a variety of highly invasive tumours [20].

It has been thought that, during the tumour metastasis, cell adhesion ability decreases, contributing to the cells dissociation from the primary site. A typical example is the reduction in the E-cadherin expression level in a variety of tumours. It was found that E-cadherin expression levels were significantly lower in ascitic and metastatic ovarian cancer cells than in the primary lesion sites of ovarian cancer, and the lower the E-cadherin expression level is, the more invasive the ovarian cancer cells are [21]. However, not all tumour metastases are related to the down-regulation of cell adhesion molecules. It is becoming increasingly clear that many cells deviated from the solid tumour in the form of tight or loose groups [22]. Therefore, it is hypothesised that the metastatic tumour cells that lack E-cadherin may be connected by other adhesion molecules to form a colony. In contrast to E-cadherin, another type of adhesion molecule, the immunoglobulin superfamily (including NCAM, MCAM, ALCAM and L1CAM, among others), is often highly expressed in metastatic tumour tissues [23].

This study focused on MCAM, a cell–cell adhesion molecule. This molecule can mediate heterotypic and homeotypic cell–cell adhesion through interaction with unknown ligands [24]. It was reported that, in mature normal tissues, MCAM is expressed mainly in endothelial cells and smooth muscle cells [25], and a certain amount is also expressed in certain activated lymphocytes and bone marrow cells [26]. Previous research showed that abnormal expression of MCAM occurs in a variety of tumours and is related to tumour development. For example, the overexpression of MCAM in melanoma cells can promote the growth and metastasis of xenograft tumours in nude mice [27]. In contrast, MCAM expression in breast cancer is reduced [28]. However, CD146 down-modulation is associated with the reversal of several biological characteristics leading to a less aggressive phenotype of breast cancer cells [10]. The fact that MCAM plays different roles in different tumours reflects the complexity of cancer molecular biology.

Our research has shown that normal ovarian surface epithelial cells do not express MCAM and that the MCAM-positive tumour ratio is very low in benign ovarian tumours. However, the MCAM-positive tumour rate significantly increased in borderline ovarian tumours and malignant epithelial ovarian tumours, suggesting that MCAM expression is correlated with tumour malignancy. It is noteworthy that the MCAM-positive rate is especially high in metastatic ovarian cancer lesions, indicating that MCAM expression may be involved in the metastasis of ovarian cancer.

Furthermore, we found that when MCAM was silenced, the growth of the ovarian cancer cell lines SKOV-3 and OVCA-429 were significantly inhibited. Becker et al. reported that reducing MCAM or beta3 integrin expression in melanoma cells by RNA interference can inhibit cell growth [29]. The specific molecular mechanisms by which MCAM affects tumour growth are not yet fully understood. We examined the cell proliferation and apoptosis and found that the apoptosis of ovarian cancer cell lines SKOV-3 and OVCA-429 were increased by silencing of MCAM. We have also found that MCAM interference in ovarian cancer cells led to a significant reduction in their in vitro invasion through Matrigel and spreading on extracellular matrix. Earlier studies have shown that the overexpression of MCAM in melanoma increased the expression of matrix metalloproteinase-2 (MMP-2), thereby contributing to the degradation of the extracellular matrix by tumour cells and promoting metastasis. Conversely, MCAM antibody blockage can down-regulate MMP-2 expression [30]. It has been shown that the expression of MMPs was regulated by small Rho GTPases (Cdc42, Rac1 and RhoA), which are involved in many normal and pathological cellular processes, including cancer invasion and metastasis [31, 32]. Small Rho GTPases were also demonstrated to be important regulators of apoptosis in both normal and tumour cells [33]. In this study, we found that the activities of Rho GTPases (Cdc42 and RhoA) were decreased by silencing of MCAM. Taken together, MCAM might regulate the Rho signalling pathway to promote ovarian cancer cell malignant invasion and metastasis and protect them from apoptosis.

In conclusion, we have demonstrated that MCAM have multiple effects on epithelial ovarian cancer cell properties, including invasion, apoptosis and spreading on extracellular matrix, which may be related to the dysregulation of small Rho GTPase (RhoA and Cdc42). In general, the inhibition of MCAM leads to a change in interaction among tumour cells and between tumour cells and the extracellular matrix, leading to the alterations in cancer invasion, metastasis and apoptosis. The findings above suggest that MCAM plays an important role in protecting epithelial ovarian cancer cell from apoptosis and promoting their metastasis, indicating that MCAM can be used as a potential target for the clinical treatment of epithelial ovarian cancer. More in-depth study will be required to clarify the value of MCAM in clinical applications.
